# Determinants of tumor treating field usage in patients with primary glioblastoma: A single institutional experience

**DOI:** 10.1093/noajnl/vdac150

**Published:** 2022-09-15

**Authors:** Matthew T Ballo, Kaitlin W Qualls, L Madison Michael, Jeffrey M Sorenson, Brandon Baughman, Saradasri Karri-Wellikoff, Manjari Pandey

**Affiliations:** Department of Radiation Oncology, West Cancer Center & Research Institute, Memphis, Tennessee, USA; Department of Radiation Oncology, West Cancer Center & Research Institute, Memphis, Tennessee, USA; Neurosurgery, Semmes-Murphey Neurosurgery Clinic, Memphis, Tennessee, USA; Neurosurgery, Semmes-Murphey Neurosurgery Clinic, Memphis, Tennessee, USA; Neurosurgery, Semmes-Murphey Neurosurgery Clinic, Memphis, Tennessee, USA; Department of Medical Oncology, West Cancer Center and Research Institute, Memphis, Tennessee, USA; Department of Medical Oncology, West Cancer Center and Research Institute, Memphis, Tennessee, USA

**Keywords:** compliance, glioblastoma, overall survival, tumor treating fields

## Abstract

**Background:**

Determinates of tumor treating fields (TTFields) usage in patients receiving combined modality therapy for primary IDH wild-type glioblastoma are currently unknown.

**Methods:**

Ninety-one patients underwent maximal debulking surgical resection, completed external beam radiotherapy with concurrent Temozolomide (TMZ), and initiated adjuvant TMZ with or without TTFields. We performed a retrospective analysis of patient, tumor, and treatment-related factors that affected TTFields usage.

**Results:**

We identified three TTFields usage subgroups: 32 patients that declined TTFields, 40 patients that started, but had monthly compliance of less than 75% or used it for less than 2 months, and 19 patients who used TTFields for 2 or more months and maintained average monthly compliance greater than 75%. With 26.5 months median follow-up for surviving patients, the 1- and 3-year actuarial overall survival for all patients was 80% and 18%, respectively. On multivariate analysis TTFields use (*P* = .03), extent of surgical resection (*P* = 0.02), and MGMT methylation status (*P* = .01) were significantly associated with overall survival. TTFields usage was explored as a continuous variable and higher average usage was associated with longer overall survival (*P* = .03). There was no relationship between patient, tumor, or treatment-related factors and a patient’s decision to use TTFields.

**Conclusions:**

No subgroup of patients was more or less likely to initiate TTFields therapy and no subgroup was more or less likely to use TTFields as prescribed. The degree of TTFields compliance may be associated with improved survival independent of other factors.

**Key Point**

 It is reasonable to offer all patients with primary glioblastoma TTFields therapy as we could not identify a group that was more or less likely to discontinue therapy or unable to initiate therapy. Patients benefit from TTFields regardless of tumor or patient characteristics.

**Importance of the study**

We hypothesized that patient or tumor characteristics would explain why some patients choose to initiate tumor treatment fields therapy for their newly diagnosed glioblastoma, while others do not. We wondered why some patients start therapy but are unable or unwilling to use the therapy as directed, while others use the therapy more than directed. Our study confirmed that tumor-treating fields may have clinical benefits in terms of survival, but we were unable to find a subgroup of patients that is more or less likely to use the therapy as directed. We suggest prescribing tumor treating fields to all newly diagnosed glioblastoma patients, but we must overcome barriers to its use so that patients who decide to start therapy maintain the recommended level of usage.

Glioblastoma is the most common and aggressive primary malignant brain tumor diagnosed in adults and has a poor prognosis, with only 5%–10% of patients being alive 5 years following diagnosis. Even with the best standard of care, consisting of maximal safe surgical resection, radiation therapy, and temozolomide (TMZ) chemotherapy, median overall survival has historically been only 14.6 months.^1,2^

Tumor treating fields (TTFields) represent a novel therapy in the treatment of glioblastoma. TTFields deliver low-intensity, intermediate-frequency (200 kHz) alternating electric fields that act as an antimitotic agent via transducer arrays applied to the scalp, selectively inhibiting the growth of rapidly dividing tumor cells.^3^ It has also been shown to disrupt multiple phases of the cell cycle, including metaphase, anaphase, and telophase, resulting in cellular apoptosis.^4,5^

In 2009, Stupp and colleagues initiated a phase 3 randomized clinical trial, EF-14, comparing postoperative TMZ chemotherapy with external beam radiation followed by monthly maintenance TMZ alone versus this same treatment regimen plus TTFields in newly diagnosed glioblastoma patients. Their results demonstrated that standard treatment plus TTFields results in longer progression-free and overall survival when compared to standard treatment alone (6.7 vs. 4.0 months and 20.9 vs. 16.0 months, respectively). This study was the first significant improvement in overall survival since TMZ was added to adjuvant external beam radiation in 2005. Further analysis of the study showed that a higher degree of TTFields usage, calculated as a percentage per month of TTFields delivery, and TTFields dose intensity, calculated as the average field intensity through the tumor bed, both independently correlated with improved survival.^6–9^

The current analysis was undertaken to provide real-world outcomes from our single institutional experience in incorporating TTFields into standard practice for newly diagnosed glioblastoma patients. We report outcomes in our cohort of patients and identify factors associated with both initiating TTFields and maintaining the required usage following initiation.

## Material and Methods

Patients were identified through the Radiation Oncology departmental brain tumor database. In 2015, we adopted a current practice protocol of treating all newly diagnosed glioblastoma patients with TTFields during the adjuvant temozolomide (TMZ) component of their therapy. We used support groups and consistent messaging across specialties to encourage all patients to initiate TTFields. Between 2015 and 2021, 135 patients diagnosed and treated for supratentorial glioblastoma were identified. We excluded patients who received best supportive care alone and patients with less than 9 months of follow up leaving a cohort of 91 patients with IDH wild-type glioblastoma who underwent maximal surgical debulking, completed external beam radiotherapy with concurrent TMZ chemotherapy, and initiated adjuvant systemic therapy. A waiver of informed consent was obtained prior to analysis from our Institutional Review Board (IRB).

Disease relapse was scored if there was any clinical or radiographic evidence of tumor regrowth and patients were followed regularly until the time of death. Actuarial data for overall survival curves were calculated using the Kaplan–Meier method and tests of significance were based on the Breslow statistic. Multivariate analysis was done with the proportional hazards model using the log-linear relative hazard function of Cox. The date of surgical resection or biopsy was used as time zero. The significance of differences between proportions was tested with the chi-square statistic or with Fisher’s exact test and differences between means were tested with the t-test or the nonparametric Mann–Whitney test as appropriate. The analysis was performed using SPSS Statistic v.28.

## Results

### Patient, Tumor, and Treatment Characteristics

Ninety-one patients underwent maximal surgical debulking, completed the prescribed dose of external beam radiotherapy (median dose 60 Gy, range 40–60 Gy) with concurrent TMZ chemotherapy, and initiated adjuvant TMZ. Seventy-four patients received 60 Gy, while 17 patients received less than 60 Gy. Patients’ ages at the time of presentation ranged from 34 to 87 years with a median of 60 years. There were 62 male and 29 female patients. Twenty-nine patients presented with frontal tumors, 22 with parietal tumors, 32 with temporal tumors, and 8 with occipital tumors. All patients underwent a complete history and physical examination, and appropriate radiological imaging studies. ECOG performance status was 0 in 54 patients,1 in 26, 2 in 8, and 3 in 3 patients. Fifty-five patients underwent gross total resection, while 25 had a subtotal resection and 11 had a biopsy only. All 91 patients had histological and molecular confirmation of WHO grade IV glioblastoma. MGMT was methylated in 43 patients, while 39 were un-methylated. MGMT methylation status could not be determined in 9 patients.

Despite strongly recommending TTFields as part of our standard regimen for patients with primary glioblastoma, not all patients accepted this recommendation as only 59 patients initiated TTFields (65%). Patient, tumor, and treatment characteristics according to TTFields use are shown in Table 1. There were no significant imbalances between patient sex, MGMT methylation status, ECOG performance status, radiation dose, and extent of surgical resection and whether the patient chose to start TTFields. Patients that chose to initiate TTFields were slightly younger than those who chose not to initiate TTFields (mean age: 59 years vs. 63 years, *P* = .05).

**Table 1.** Characteristics of the Patients by TTField Use, %

Characteristic	No TTFields use	TTFields use	*P*-value
Mean Age, years			NS
≤60y	26	74	
>60y	43	57	
Sex			NS
Male	32	68	
Female	41	59	
MGMT promoter status			NS
Methylated	35	65	
Un-methylated	33	67	
ECOG Group			NS
0–1	34	66	
2–4	46	54	
Type of surgery			NS
Gross total resection	31	69	
STR/Biopsy only	42	58	
Radiation dose			NS
60 Gy	32	68	
<60 Gy	47	53	

Abbreviation: MGMT, O^6^-methylguanine–DNA methyltransferase; ECOG, Eastern Cooperative Oncology Group; STR, Subtotal resection; NS, not significant.

**Figure 1.**
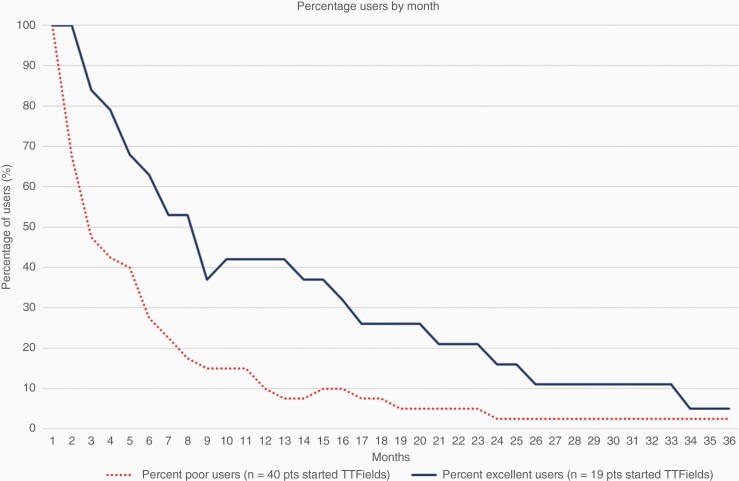
Percentage of users continuing to use TTFields each month after initiating therapy.

**Figure 2.**
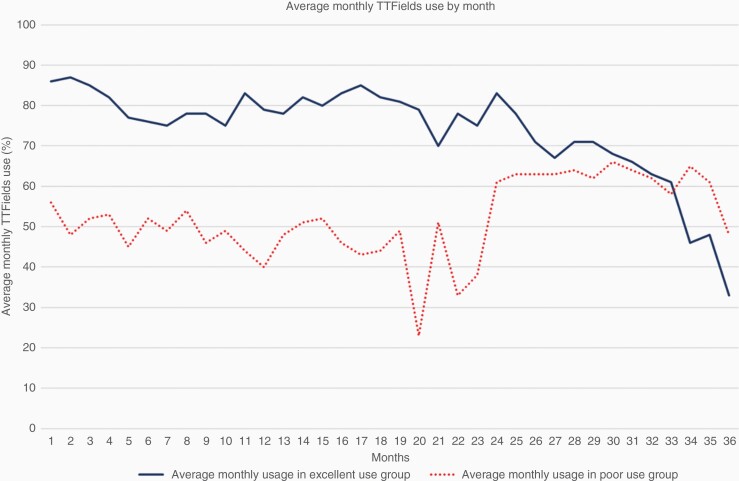
Average monthly use at each month after initiating TTFields.

As part of our standard regimen, we strongly encouraged patients to use TTFields ≥18 h per day (equivalent to average monthly compliance of ≥75%) as the randomized clinical trial suggested that this was associated with improved overall survival.^6–8^ Monthly TTFields usage data were collected on each patient, and we identified three TTFields usage subgroups within our cohort: In total 32 patients that declined TTFields altogether (no use group), 40 patients that started, but had monthly compliance of 1%–75% or used it for less than 2 months (low use group), and 19 patients who used TTFields for 2 or more months and maintained an average monthly usage greater than 75% over their first 3 months of use (high use group). Within the low-use group, the number of months used ranged from 1 to 44 months (median, 3 months) with average monthly compliance ranging from 9% to 87% (median, 57%, SD 22%). Within the high-use group, the number of months used ranged from 2 to 38 months (median, 9 months) with average monthly compliance ranging from 75% to 96% (median 84%, SD 6%). Figures 1 and 2 visually present the usage among the low users and the high users. Figure 1 shows the percentage of users who continue to use TTFields each month after initiating therapy and Figure 2 shows the average monthly usage each month after initiating therapy. To determine any special characteristics of this highly motivated group of 19 patients, we examined patient, tumor, and treatment characteristics among these 3 distinct groups of patients (Table 2). There were no significant imbalances between patient age, sex, MGMT methylation status, ECOG performance status, radiation dose, or extent of surgical resection and whether the patient used TTFields for 2 or more months and maintained a 75% average monthly compliance for their first 3 months of use (ie, being a high user).

**Table 2.** Characteristics of the Patients by Extent of TTField Use, %

Characteristic	No TTFields use	Low TTFields use	High TTFields use	*P*-value
Mean age, years				NS
≤60y	26	52	21	
>60y	43	37	20	
Sex				NS
Male	32	47	21	
Female	41	38	21	
MGMT promoter status				NS
Methylated	35	42	23	
Un-methylated	33	49	18	
ECOG Group				NS
0–1	34	45	21	
2–4	46	36	18	
Type of surgery				NS
Gross total resection	31	49	20	
STR/Biopsy only	42	36	22	
Radiation dose				NS
60 Gy	32	45	23	
<60 Gy	47	41	12	

Abbreviation: MGMT, O^6^-methylguanine–DNA methyltransferase; ECOG, Eastern Cooperative Oncology Group; STR, Subtotal resection; NS, not significant.

### Patient Survival

The median duration of follow-up for the 18 patients alive at last contact was 26 months (range, 10 to 66 months). The actuarial 1-, 2-, and 3-year overall survival rates for all patients were 80%, 33%, and 18%, respectively. Table 3 shows the 2-year actuarial rate of overall survival according to patient, tumor, and treatment characteristics. TTFields use (not used, 15 months median OS vs. used, 20.7 months OS, *P* = .04), extent of TTFields usage (not used, 15 months median OS vs. low usage, 20 months median OS vs. high usage, 28 months median OS, *p* = .05), age (>60, 16 months median OS vs. ≤60, 22 months median OS, *P* = .03), extent of surgical resection (subtotal resection/biopsy only, 16 months median OS vs. gross total resection 21 months median OS, *P* = .04) and MGMT methylation status (un-methylated, 16 month medians OS vs. methylated, 22 months median OS, *P* = .04) were all significant on univariate analysis.

**Table 3.** Analysis of Characteristics Potentially Affecting 2-Year Actuarial Survival

Characteristic	*n*	% OS	*P*-value
Sex			NS
Male	62	32	
Female	29	34	
Mean age, years			.03
≤60y	42	41	
>60y	49	26	
MGMT promoter status			.04^†^
Methylated	43	44	
Un-methylated	39	20	
ECOG			NS
0–1	80	34	
2–4	11	24	
Type of surgery			.03^†^
Gross total resection	55	37	
STR/Biopsy only	36	25	
TTField use			.04^†^
No	32	29	
Yes	59	35	
Extent of TTFields use			.05^†^
No use	32	29	
Low use	40	27	
High use	19	54	

^†^ Remained significant on multivariate analysis.

Abbreviations: OS, Overall survival; NS, not significant; STR, Subtotal resection; ECOG, Eastern Cooperative Oncology Group.

**Figure 3.**
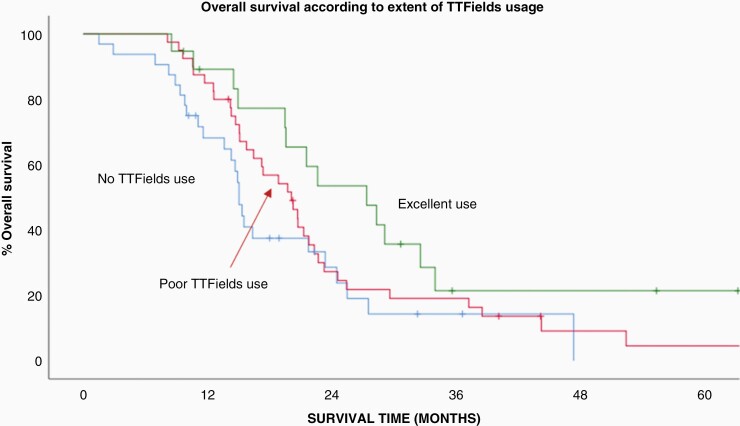
Actuarial survival curves according to the extent of TTFields usage (*P* = .04).

To control for any imbalance between important prognostic factors a multivariate analysis was performed (Table 3). This revealed that TTFields use (*P* = .03), extent of surgical resection (*P* = .02) and MGMT methylation status (*P* = .01) remained independently significant. When the extent of TTFields use (not used vs. low use vs. high use) was entered into the model instead of TTFields use (yes vs. no) it was also independently predictive of overall survival (*P* = .05). The actuarial survival curves according to the extent of TTFields usage is shown in Figure 3. We also investigated TTFields use as a continuous variable and found that overall survival was associated with the TTFields usage expressed as the average monthly usage over the first 3 months of use (multivariate *P* = .03).

## Discussion

We present our institutional analysis of outcomes for newly diagnosed IDH wild-type glioblastoma patients who utilized TTFields in a nonclinical trial setting. We report that TTFields use may be associated with improved overall survival and a higher degree of use may be associated with improved overall survival independent of another patient, tumor, or treatment factors.

We initiated our current practice protocol in 2015 and soon recognized that there were three distinct groups of patients. Some patients immediately refused to initiate TTFields therapy while others were so enthusiastic about starting that they voiced reluctance to wait for the prescribed 4-week break between concurrent TMZ/radiation and the initiation of adjuvant TMZ/TTFields. Somewhere in the middle was a third group of patients unable to or unwilling to fully commit to the therapy, but wanting to start, nonetheless.

Given the results of EF-14 and subsequent post hoc analyses, we wanted all patients to not just start TTFields but to commit to greater than 2 months of use and greater than 75% monthly compliance. Despite our best efforts, 3 groups remained, and the current analysis was intended to help us identify who might fall into one group versus another and how we might affect individual patient decisions. Unfortunately, we are unable to identify any specific patient, tumor, or treatment-related characteristics that correlate with TTFields use. Radiation dose, for example, was examined because the dose is a surrogate for ECOG performance status, patient age, and extent of disease as we traditionally dose reduce to 57Gy in 30 fractions for patients with very large volumes of disease and utilize 40Gy in 15 fractions for patients who are older or have a lower performance status.^10,11^ Although we expected this patient subset to make up the majority of the non-TTFields users, with potentially worse baseline performance status, older age, and larger tumors, there was no difference major in TTFields use between groups. A similar lack of correlation was seen for all the patient, tumor, and treatment-related characteristics studied (Table 1).

It is possible that some other factor more difficult to define or extract from a retrospective chart review determines one’s decision to use or not use TTFields. Factors such as home environment, social support, cognitive function, level of education, or combinations of these factors are almost certainly involved in decision-making and will be topics of further study. We did eliminate individual healthcare providers and their communication skills as at least one variable as we made the use of TTFields part of our standard of care where each member of the care team restated the importance of its use. Despite our coordinated messaging and delivery of care we still had 35% of patients refuse to even begin TTFields despite proceeding with adjuvant systemic therapy.

For the purposes of categorical analysis, we defined our high-use group as those who used the device for at least 2 months and maintained average monthly compliance greater than 75% over their first 3 months of use. The greater than 75% use was chosen to match that required in the EF-14 randomized trial.^6,8^ Our definition was carefully chosen to give patients just over 1 month to become accustomed to the device and determine how best to achieve compliance above 75%. Any other definition that includes longer periods of usage as a criterion for entry would bias the results as patients who live longer are the same patients that can use the device for a longer period. To avoid selection bias, we assumed that no patient needs more than 2 months to make a commitment to TTFields use.

After a median follow-up of 26 months, patients who used the device as prescribed (ie, the high use group) had a median overall survival of 28 months. This was compared favorably to the group that was low users (20 months) and the group that chose not to use TTFields at all (15 months, *P* = .05) which is illustrated in Figure 3. This result is consistent with that reported by Toms et al. who performed a secondary analysis of EF-14 trial patients.^7^ They categorized patients into groups according to their level of monthly usage and reported a stepwise improvement in overall survival with progressively increasing use. For their patients with a usage rate of less than or equal to 30%, 30%–50%, 50%–60%, 60%–70%, 70%–80%, 80%–90%, and greater than 90% the median overall survival increased from 18.2, 17.9, 18, 19.9, 21.7, and 21.5 to 24.9 months, respectively. These investigators determined a threshold of 50% average usage as the minimum value necessary to improve overall survival and that maximal improvement was seen for patients with monthly usage greater than 90%. Stupp et al. also looked at the degree of TTFields usage and reported that the 265 patients in EF-14 that were treated with TTFields for 18 h a day or more (defined as the monthly average in the first 6 months of treatment) had longer survival than the 185 patients treated less than 18 h a day (22.6 months vs. 19.1 months, *P* = .009).^6^ Viewed within the context of our result, the data strongly suggests that TTFields use and its relationship to overall survival is directly proportional and that for every hour of additional use there is an improvement in overall survival.

So, what then to do with the results of our analysis? We have confirmed in a nonclinical trial setting that TTFields may improve overall survival and the magnitude is clinically relevant enough that we detected a dose–response effect (ie, higher usage leads to higher overall survival) in a relatively small number of patients. However, we were unable to answer the commonly posed question of who exactly should and who should not use TTFields. For the time being, the answer appears to be that every newly diagnosed glioblastoma patient should be offered TTFields therapy and there is no subgroup more likely to decline to start or more prone to premature discontinuation of therapy. The focus then should be on educating and motivating patients to use the device rather than trying to identify patients that will not benefit.

Real clinical improvements in overall survival will only be seen if we effectively overcome patient-related or physician-related barriers to TTFields usage. All patients who elect to use TTFields receive monthly follow-ups from a company-sponsored device specialist and have access to a phone-based hotline should they experience any nonclinical issues. Patients are also seen in follow-up every 2–3 months by their primary oncologist and have clinical support available to them should any issues arise. Caregiver barriers, however, may be harder to overcome as some patients simply do not have the home support system necessary to implement TTFields use. For these patients more robust clinic-based support groups might be effective, and for a minority of patients, TTFields use may simply be impossible. Opportunities for improved usage will come from either increasing the number of months a patient uses the device or by increasing daily usage, which ultimately translates into increased average use per month. After 6 months of device usage, only 30% of our low-use group were still using the device versus almost 70% of the high-use group (Figure 1). Also, over the entire period of device usage measured, the high use group started high and maintained a high use, while the low use group started low and maintained a low use (Figure 2). Future endeavors include working to maximize support availability for all patients and addressing any barriers to utilization that may exist, but primarily need to focus on the low use group that starts with low use and quickly discontinues treatment.

Possibly even harder to overcome are ingrained institutional barriers. For example, academic centers with funded non-TTFields research might view patients’ TTFields use as interfering with institutional protocols. While this is certainly a practical issue with data analysis this view represents a clear conflict of interest between institutional funding and improved patient outcome. Overcoming these types of barriers can only occur through patient advocacy and working with academic centers to design trials upon the strongest clinical foundation possible. To immediately address this barrier, TTFields use should not be an exclusion criterion for protocol enrollment.

## Conclusions

Our results complement randomized trial data in that TTFields use may lead to improved overall survival and a higher degree of use may improve overall survival independent of another patient, tumor, or treatment factors. No subgroup of patients was more or less likely to initiate TTFields therapy and no subgroup was more or less likely to use TTFields as prescribed. Efforts should focus on improving individual patient TTFields use and overcoming barriers to TTFields use.

## Data Availability

Data are not available currently.
